# Ensuring healthcare workers’ safety in the management of Ebola virus disease: a novel competency assessment checklist for proper PPE use

**DOI:** 10.1186/2047-2994-4-S1-P6

**Published:** 2015-06-16

**Authors:** E Tartari, AR Falzon Parascandalo, MA Borg

**Affiliations:** 1 Infection Control Unit, Mater Dei Hospital, MSIDA, Malta

## Introduction

In response to EVD outbreak in Africa, Malta like many other countries in Europe, stepped up efforts to prepare and train in correct procedures for safe identification and management of potential EVD patients in the main acute tertiary hospital.

## Objectives

We wanted our training to not only provide theoretical knowledge but, generate competence and ensure staff would be able to manage a case, and avoid the situation reported in western hospitals where inadequate proficiency in PPE use was identified as the cause of cross-infection in HCWs.

## Methods

Infection control specialists in collaboration with the infectious diseases department, developed a comprehensive training programme for identified core team members. We developed new methods, focused around a 'PPE donning and doffing competency assessment checklist', constantly monitored by a buddy and a CCTV control system. Simulation technology was utilized through performing weekly drill exercises. Daily PPE hands-on training and demonstration were conducted (August-October 2014) in the high containment isolation unit. Each individual had the opportunity to train for more than 10 sessions. Following training, formal competency assessments were conducted for 55 HCWs (November-January 2015) . A 100% compliance was necessary to be declared as competent.

## Results

The checklist proved to be an important novel method ensuring the preparedness of HCWs when managing patients suspected for EVD. The sequence of buddy-assisted doffing procedure was identified as the most complicated, where HCWs generated the majority of the failing points, and necessitating more than one assessment. This exercise highlighted existing uncertainties and inconsistencies in PPE sequence, despite previous extensive training. In these situations, retraining of the HCW was undertaken and assessment repeated until a satisfactory result was obtained.

## Conclusion

The PPE competency assessment method was found to be a useful tool in harmonizing the training provided with actual performance in clinical practice. The checklist provided a systematic approach to easily identify HCW's conformances and non-conformances with the protocol. This method can easily be developed for other clinical procedures in the healthcare setting, even in backgrounds of limited resources.

## Disclosure of interest

None declared.

